# Periodicity in Attachment Organelle Revealed by Electron Cryotomography Suggests Conformational Changes in Gliding Mechanism of *Mycoplasma pneumoniae*

**DOI:** 10.1128/mBio.00243-16

**Published:** 2016-04-12

**Authors:** Akihiro Kawamoto, Lisa Matsuo, Takayuki Kato, Hiroki Yamamoto, Keiichi Namba, Makoto Miyata

**Affiliations:** a Graduate School of Frontier Biosciences, Osaka University, Suita, Osaka, Japan; b Department of Biology, Graduate School of Science, Osaka City University, Sumiyoshi-ku, Osaka, Japan; c Riken Quantitative Biology Center, Suita, Osaka, Japan; d The OCU Advanced Research Institute for Natural Science and Technology (OCARINA), Osaka City University, Sumiyoshi, Osaka, Japan

## Abstract

*Mycoplasma pneumoniae*, a pathogenic bacterium, glides on host surfaces using a unique mechanism. It forms an attachment organelle at a cell pole as a protrusion comprised of knoblike surface structures and an internal core. Here, we analyzed the three-dimensional structure of the organelle in detail by electron cryotomography. On the surface, knoblike particles formed a two-dimensional array, albeit with limited regularity. Analyses using a nonbinding mutant and an antibody showed that the knoblike particles correspond to a naplike structure that has been observed by negative-staining electron microscopy and is likely to be formed as a complex of P1 adhesin, the key protein for binding and gliding. The paired thin and thick plates feature a rigid hexagonal lattice and striations with highly variable repeat distances, respectively. The combination of variable and invariant structures in the internal core and the P1 adhesin array on the surface suggest a model in which axial extension and compression of the thick plate along a rigid thin plate is coupled with attachment to and detachment from the substrate during gliding.

## IMPORTANCE

Human mycoplasma pneumonia, epidemic all over the world in recent years, is caused by a pathogenic bacterium, *Mycoplasma pneumoniae*. This tiny bacterium, about 2 µm in cell body length, glides on the surface of the human trachea to infect the host by binding to sialylated oligosaccharides, which are also the binding targets of influenza viruses. The mechanism of mycoplasmal gliding motility is not related to any other well-studied motility systems, such as bacterial flagella and cytoplasmic motor proteins. Here, we visualized the attachment organelle, a cellular architecture for gliding, three dimensionally by using electron cryotomography and other conventional methods. A possible gliding mechanism has been suggested based on the architectural images.

## INTRODUCTION

Human mycoplasma pneumonia, so-called walking pneumonia, has caused periodic global epidemics (1, 2). The causative bacterium, *Mycoplasma pneumoniae*, forms a protrusion at one cell pole and exhibits gliding motility in the direction of the protrusion (see Video S1 in the supplemental material) (3–6). The maximum speed reaches 1 µm (half of the cell length) per second (7–9). This motility, combined with the ability to adhere to epithelial cells, is involved in the parasitism of the organism, enabling the cells to translocate from the tips of the bronchial cilia to the surface of the host cell (10, 11). Previous studies, including genome analyses, have shown that this motility is not related to other known mechanisms of bacterial movement, nor does it involve motor proteins known to be involved in eukaryotic cell motility (5, 12, 13). The protrusion is called an attachment organelle or a tip structure, and this organelle is also responsible for gliding movement (4, 8, 14).

Electron cryotomography (ECT) showed that the attachment organelle contains knoblike surface protrusions and an internal core (15, 16). The internal core can be divided into three parts: a terminal button, a pair of thin and thick plates, and a bowl complex, which is also called a wheel complex, aligned in this order from the leading edge (front) of the cell. Recent studies have shown that mycoplasmas repeatedly catch and release sialylated oligosaccharides (SOs) fixed on solid surfaces (17–19). To date, 15 proteins have been identified as components of the attachment organelle and mapped onto the image systematically (20). Although their functions cannot be inferred from their amino acid sequences, many of these components are essential for adhesion and gliding motility, showing that the whole organelle functions in a coordinated manner (4, 6). Elucidation of the gliding mechanism based on the coordinated behaviors of proteins requires information on the alignment and the stability of these proteins.

One component protein, the P1 adhesin, is a 170-kDa protein with an amino acid sequence composed of three predicted domains and a single transmembrane segment; its dimer forms a heterotetramer complex with a dimer of another protein, P90 (21–24). This P1 adhesin complex has been suggested to function as the leg in the gliding mechanism, because the gliding speed of *Mycoplasma* is reduced in the presence of a monoclonal antibody against P1 adhesin (14). The P1 adhesin complex may correspond to the surface nap structure observed by negative-staining electron microscopy (EM), because they show the same subcellular localization (21, 22, 24–27).

In the present study, we analyzed the attachment organelle of the wild-type strain and a mutant lacking P1 adhesin in detail, using ECT, immuno-EM, and negative-staining EM, and visualized the two-dimensional array of the P1 adhesin complex, which has a limited degree of regularity, and the periodic structures of the paired plates. The results suggest that the thick plate may elongate and compress along the rigid thin plate, coupling its movement with P1 adhesin alignment in the gliding mechanism.

## RESULTS

### Structural features of the attachment organelle.

**FIG 1:**
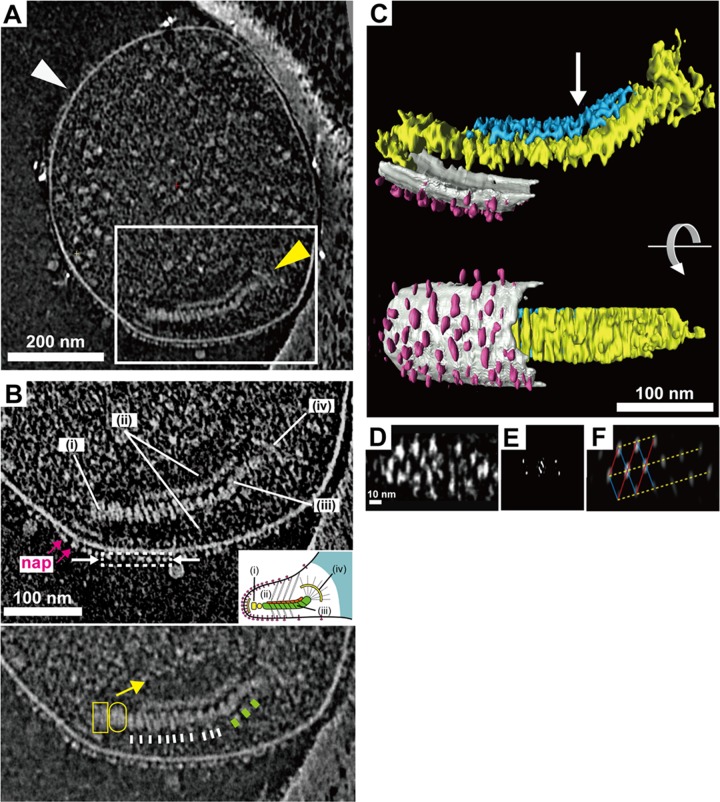
Attachment organelle image reconstituted from ECT. (A) Images of 15-nm-thick slices through the tomograms of wild-type cells. The attachment organelle can be observed in the white box. The whole cell was flattened in a thin film of ice. (B) Image of a 16-nm-thick slice through the tomogram of an attachment organelle. Top: the image shows a dense array of the knoblike particles on the membrane surface (nap) and the terminal button (i), translucent area (ii), paired plates (iii), and bowl (wheel) complex (iv). Inset: schematic diagram of the attachment organelle. Bottom: identical to the image above but at lower contrast. The outline of the translucent area is marked with a yellow arrow. The thick and thin striations of the thick plate are marked by white and green bars, respectively. The terminal button is marked by a box and an oval corresponding to those in the inset schematic (i). (C) Segmented three-dimensional image of the tomogram shown in panel A depicting the knoblike particles (pink), membranes (light gray), and thin (blue) and thick (yellow) plates of the internal core. The bend is marked with an arrow. The slices through the tomogram, the segmentation, and the rotation can be seen in Video S2 in the supplemental material. (D) Face-on view of the knoblike particles extracted from the boxed area indicated with the dashed line in panel B, with a slice thickness of 1.2 nm. (E) Fourier transform of the view in panel D. (F) Noise-filtered image of the view in panel D. The lattice lines (blue, red, and yellow) are indicated.

We analyzed the attachment organelle by ECT. Although the organelle usually has a tapered shape under the optical microscope, this feature was lost in most of the frozen-hydrated cells on EM grids prepared for ECT (Fig. 1). This was most likely due to the surface tension that the solution exerted on the cell membrane during the process of blotting to generate a thin film of solution. In the process, the cells were frozen in the thin film of vitreous ice within each miniscule hole of the porous carbon grid. However, since each organelle had an internal core within intact cells, we could pinpoint the location of the organelle by using the internal core as a marker.

Even though the tapered shape of the attachment organelle was lost due to the surface tension exerted by the ice during the blotting and freezing process, the three-dimensional images reconstructed by ECT revealed the structural features of the organelle, including the terminal button, the paired thick and thin plates, the bowl, and the knoblike particles on the surface (Fig. 1B and C; see also Video S2 in the supplemental material). The thick plate features notable striations (marked by short bars in Fig. 1B, bottom).

The central part of the internal core is a pair of thin and thick plates that are both elongated along the long axis of the core (Fig. 1C). The thin plate (Fig. 1C, blue) was 10.6 ± 1.5 nm thick (mean ± standard deviation; *n* = 30), 43.5 ± 7.1 nm wide (*n* = 11), and 106.2 ± 11.4 nm and 51.8 ± 5.6 nm long (*n* = 23) for the leading and trailing parts, respectively, on either side of the bend. The thick plate (Fig. 1C, yellow) was 22.3 ± 4.3 nm thick (*n* = 30), 43.5 ± 7.1 nm wide (*n* = 11), and 122.4 ± 10.6 nm and 58.5 ± 10.2 nm long (*n* = 23) for the front and back parts, respectively, on either side of the bend. The thick plate was clearly segmented along its long axis, with a periodicity of around 8 nm. These observations are consistent with those of previous studies (15, 16, 20, 28).

A translucent area with an absence of large cytoplasmic particles, such as ribosomes, was also clearly observed for 25 to 30 nm on both sides of the internal core along its length (Fig. 1B), as described previously (15, 16, 28). This area could be occupied by less diffusive materials than the other part of the cytosol, which may be blocking the entrance of cytoplasmic particles into this area, but their structures are too small or too thin to show good contrasts for detection in the ECT image.

### Surface structures of the attachment organelles of wild-type and mutant strains.

As cytadherence and gliding occur at the interface between the surface of the mycoplasma cell and solid surfaces, we focused on the detail of the surface structure of the attachment organelle. We found knoblike particles clustering on the surface, as previously reported (15, 16). To visualize the alignment of these particles on the membrane surface, we extracted the layer containing the knoblike particles, which are 7 to 8 nm from the membrane surface (Fig. 1B, dashed rectangle), from the tomograms to obtain a view perpendicular to the membrane (Fig. 1D) and then calculated the Fourier transforms of the images of this layer to examine the periodicity (Fig. 1E). The elongated shape of the knoblike particles in the vertical direction, as shown in Fig. 1C and D, is an artifact created by the missing wedge effect caused by the limit of tilting of the specimen (70°). The Fourier transform revealed a few well-defined spots, and the noise-filtered image generated by back Fourier transform revealed some features of a regular rhombic lattice (Fig. 1F). This suggested that the two-dimensional array of the knoblike particles had a somewhat regular periodicity, but the distances between the neighboring knoblike particles varied, ranging from 16 to 26 nm. Therefore, the regularity is limited to a local area, extending only to the second neighbors at most (Fig. 1C and D).

**FIG 2:**
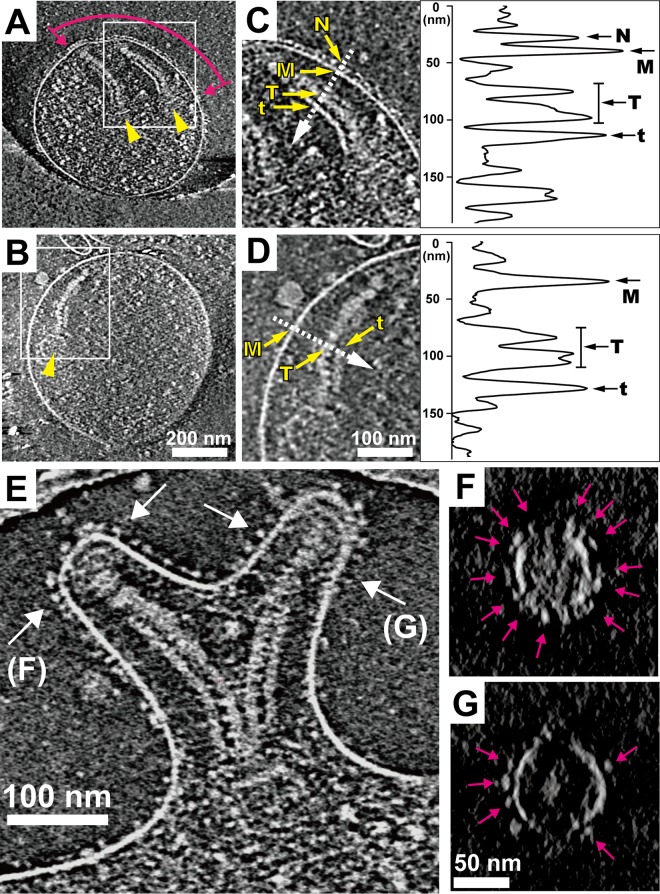
Analysis of the organelle surface by ECT. (A, B) Images of 15-nm-thick slices through the tomograms of wild-type (A) and class IV-22 mutant (B) cells. The extent of the distribution of knoblike particles on the membrane surface is indicated by a pair of magenta arrows. The internal core of the attachment organelle is indicated by yellow arrowheads. (C, D) Magnified images of the internal core of a wild-type cell (C) and a class IV-22 mutant cell (D), respectively. The yellow arrows indicate the knoblike particle (N), cell membrane (M), thick plate (T), and thin plate (t) of the internal core. Density profiles across the membranes of the wild-type cell and the class IV-22 mutant cell (dashed-line arrows) are shown in the panels to the right. (E) A 15-nm-thick slice through the tomogram of a bifurcated attachment organelle. (F, G) Cross sections of the tomogram in panel E at the positions indicated by the two pairs of arrows labeled F and G; the slices are 18 nm thick. The pink arrows indicate the knob domains of the P1 adhesin complexes on the membrane surface. For the view in panel G, a series of cross sections through the axis of protrusion can be seen in Video S3.

A nap structure observed by negative-staining EM has been suggested to correspond to the P1 adhesin complex (21, 22, 24, 27). The knoblike particles observed by ECT may correspond to P1 adhesin and the nap, but this has not been examined (15, 16). Therefore, we applied ECT to the surface of the attachment organelle of the wild-type cells, as well as the cells of the class IV-22 mutant, which is known to lack both P1 adhesin and nap due to the frameshift mutation in the gene MPN141 encoding P1 adhesin (24, 29). In the wild-type cells, the knoblike particles were found on the membrane surface in proximity to the internal core in more than 30 organelles examined (Fig. 2A). The knob-shaped morphology and dimensions of individual particles on the membrane surface were in good agreement with those described in previous ECT studies (Fig. 1C and 2) (15, 16). The knoblike density was about 6 nm in diameter, with its center located about 10 nm away from the middle of the cell membrane. Because the lipid bilayer was 5 nm thick, the center of the knob domain was 7 to 8 nm from the membrane surface, and the stalk connecting the knob to the membrane was 4 to 5 nm long (Fig. 2A and C). The knoblike particles were distributed over a distance of 360 ± 60 nm (*n* = 5) on the membrane surface, codistributed with the internal core (Fig. 2A). The knoblike particles were not observed at all on the membrane surface of the class IV-22 mutant cells (Fig. 2B and D). These observations suggest that P1 adhesin is the protein that forms the knoblike particles. The internal structures, including the internal core and the translucent area, closely resembled those of the wild-type strain, consistent with the results of a previous study (25), suggesting that the P1 adhesin complex is not involved in the formation of internal structures.

During ECT data collection from more than 30 frozen cells on grids, we found only one cell with a bifurcated tapered protrusion, in which two internal cores were connected at their proximal ends and shared a bowl structure. The thin and thick plates were both apposed to the membrane at a similar distance of about 25 nm. Such connected cores have been suggested to be the intermediates in preparation for cell division (7, 20, 26, 30), and the morphology of the paired cores in this bifurcated cell protrusion appeared to correspond to this intermediate stage. The protrusion was about 200 nm long, and cross sections at the planes indicated by the pairs of arrows labeled F and G in Fig. 2E revealed a slightly elliptical membrane shape with diameters of 55 and 70 nm for the slices shown in Fig. 2F and G, respectively. The knoblike particles were found all around the tapered membrane protrusion, as observed in the images in Fig. 2F and G, as well as in Video S3 in the supplemental material. The two-dimensional arrays of knoblike particles on the surface of the protrusions and the core structures were similar to those observed in other tomograms of the organelle, where the tapered shape was lost and the membrane was flattened, suggesting that the structural features of the nap and core were not markedly distorted by the flattening of the cells.

### P1 adhesin and the nap structure in wild-type and class IV-22 mutant cells.

**FIG 3:**
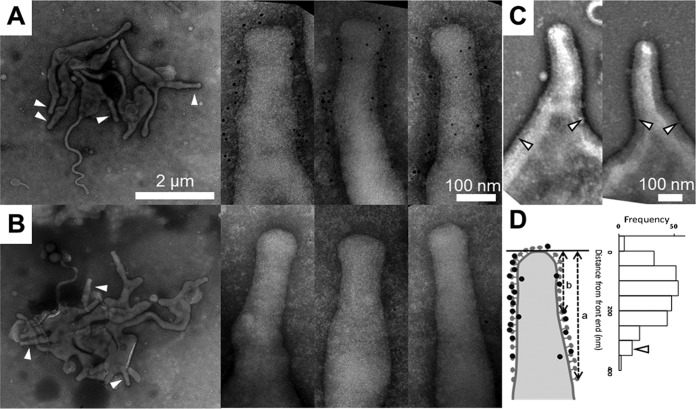
Distribution of the P1 adhesin complex on negative-staining EM images of *Mycoplasma pneumoniae* cells. (A) Immuno-EM showing P1 adhesin distribution on wild-type cells. A field image and magnified images are shown in the leftmost and remaining three panels, respectively. White arrowheads indicate the attachment organelle. The position of the P1 adhesin can be identified by a 5-nm black dot, which is a colloidal gold particle conjugated to an anti-P1 adhesin monoclonal antibody. (B) Control experiments on class IV-22 mutant cells lacking P1 adhesin, P90, and P40 proteins. (C) Negative-staining EM of the attachment organelle showing the nap structures on the membrane surface. The limits of the regions of nap distribution are marked with open arrowheads. (D) Left: definition of the region limit of continuous nap distribution (a) and the positions of colloidal gold particles (b) measured from the front end of the cell. Right: distribution frequencies of black dots found around the cell protrusion, representing the distribution of the P1 adhesin complex along the cell axis. The average of the limits of nap arrays from the front end of the cell is indicated with an open arrowhead. The data collected from 10 cell images were integrated.

We conducted immuno-EM, using a monoclonal antibody specific for the P1 adhesin, and negative-staining EM to clarify whether the knoblike structures, P1 adhesin, and nap structure were identical (Fig. 3) (14). By immuno-EM, the mycoplasma cells were 1,350 ± 140 nm (*n* = 10) long and featured a membrane protrusion at a cell pole, as revealed by previous studies; this was the attachment organelle. Gold particles labeling P1 adhesin were found frequently, mostly located around the surface of the organelle, and were distributed over a region about 350 nm from the front end, with a distribution peak at 150 ± 70 nm (*n* = 10 cells) (Fig. 3D). This was in agreement with the length of the nap distribution observed by ECT (Fig. 2), as well as with the results of our previous immunofluorescence studies (25, 26). The average number of gold particles was 28 ± 5 per cell (*n* = 10). We also performed immuno-EM on a mutant strain, class IV-22, and found only a few gold particles per cell without any specific localization, confirming that this strain lacks P1 adhesin (Fig. 3B).

To examine the distribution of the nap structure on the membrane surface, we observed the cells by EM with simple negative staining, because the procedure for immuno-EM reduces the image contrast (Fig. 3C). The nap structures were localized over a region 330 ± 40 nm (*n* = 12) from the front end, which corresponded well to the localization of the gold particles labeling the P1 adhesin (Fig. 3D). These results indicated that the P1 adhesin complexes corresponded to the nap structures observed by negative-staining EM and also to the knoblike structures observed by ECT.

### Regular arrays within the thin and thick plates.

**FIG 4:**
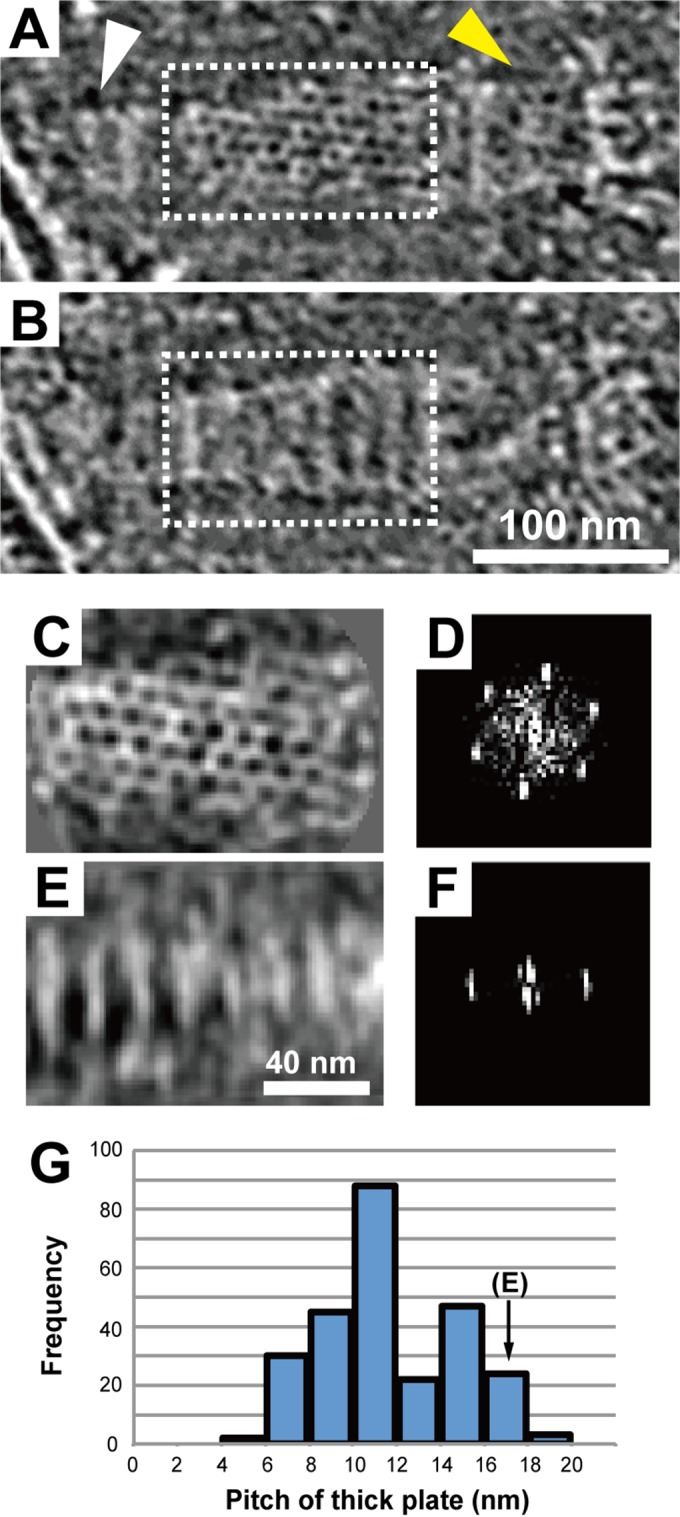
Regular two-dimensional lattice structures of the thin and thick plates. (A, B) Two 12-nm-thick slices through a tomogram, separated by 20 nm, showing the face-on views of the thin and thick plates as highlighted with dashed-line boxes. The terminal button and the bowl (wheel) complex are marked by white and yellow arrowheads, respectively, in panel A. A series of slices of this tomogram is available as Video S4 in the supplemental material. (C, D) An averaged image of the thin plate (*n* = 8) and the image of its Fourier transform. A nearly hexagonal lattice was obtained by averaging 10 independent images of the thin plate. (E, F) An averaged image of the thick plate and the image of its Fourier transform. Images of the thick plate were averaged for independent images with periodicities ranging from 16 to 18 nm (*n* = 4). The image shows a segmented structure with periodicity along the long axis of the plate. (G) Distribution of thick plate pitches computed from the images of their Fourier transforms. The pitch of the thick plate in panel E is marked.

Next, we analyzed the structures of the paired plates. Images of consecutive slices of the internal structures clearly revealed periodic structural patterns for the thin and thick plates (Fig. 4A and B; see also Video S4 in the supplemental material). The averaged image of the thin plates in a face-on view revealed a regular two-dimensional array of a nearly hexagonal lattice (*n* = 8) (Fig. 4C). The Fourier transform revealed well-defined spots, confirming the regular lattice structure (Fig. 4D). One of the lattice lines is tilted about 7° from the axis of the thin plate. The lattice spacing was measured on eight tomograms showing the face-on view of the thin plate, and it was found to be 7.5 ± 0.5 nm. The standard deviation of less than 10% suggested that the thin plate was an invariable structure.

The two-dimensional array within the plane of the thick plate was not as clear, but the periodic segmentation along the long axis was obvious in many tomograms (Fig. 4B and E). Fourier transform also indicated the presence of a one-dimensional array (Fig. 4F). The segmentation lines were mostly perpendicular to the long axis of the thick plate. Interestingly, the periodicity of the thick plate was much more variable than that of the thin plate, both within individual samples and between samples, ranging from 4 to 20 nm (Fig. 4G), with a mean and standard deviation of 10.4 ± 3.2 nm (*n* = 26).

**FIG 5:**
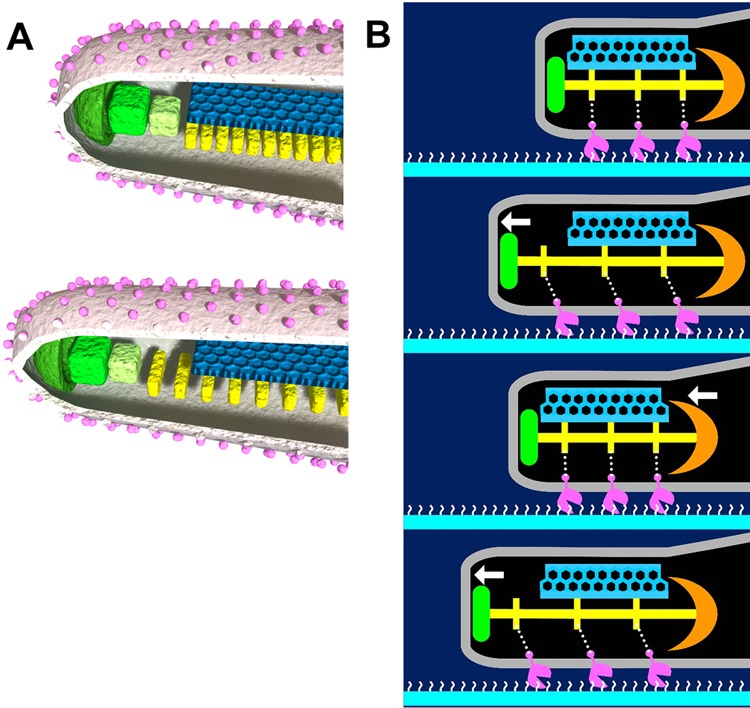
(A, B) Novel features of the machinery (A) and suggested mechanism (B) underlying gliding. (A) The P1 adhesin molecules, colored pink, are present in a rhombic lattice with limited regularity. The thin and thick plates, colored blue and yellow, respectively, feature a rigid hexagonal lattice and striations with highly variable pitches, respectively. The compression and extension of the thick plate may cause shortening (upper) and elongation (lower), respectively, of the cell protrusion. The terminal button is marked in green. An animation of these images is shown in Video S5 in the supplemental material. (B) The thick plate changes its pitch and induces directed detachment of P1 adhesin from SOs, colored white, resulting in elongation of the cell protrusion and leading to cell displacement from right to left. See the text for details. This suggested mechanism is also demonstrated in Video S6.

The findings from the present study are summarized in Fig. 5A.

## DISCUSSION

### ECT of the attachment organelle

In previous studies, the attachment organelle of *M. pneumoniae* was analyzed three-dimensionally by ECT (15, 16). However, the fine structures of the core, including regularities and periodicities, have not previously been analyzed in detail, although it was known that the thick plate featured striations. In the present study, to analyze these periodicities, we embedded the cells in thin films of ice and collected ECT data with higher angular ranges and finer intervals in a tilt series and using a more sensitive camera than in previous studies. Based on these improved images, we here describe (i) a two-dimensional array of the knoblike particles on the surface, (ii) the hexagonal lattice of the thin plate, and (iii) the actual variation in pitches of the thick plate. In previous ECT studies, the array of the knoblike particles and the periodic two-dimensional lattice of the thin plate had not been observed (15, 16). Although the variation in the pitch of the thick plate was mentioned, this variation was not quantitatively analyzed (15, 16, 28).

### Correspondence of P1 adhesin complex and nap with knoblike particles.

Although the P1 adhesin complex localizes to the surface of the cell and is essential for cytadherence and gliding (14, 22, 23, 29), it has not been identified in ECT images of mycoplasma cells. Previous studies showed that the distribution of the nap structure observed by negative-staining EM agreed with that observed by immuno-EM for P1 adhesin (22, 24, 27). However, the relation between the knoblike particles observed by ECT and either the nap in negative-staining EM images or P1 adhesin has not been examined. Moreover, identity between the P1 adhesin complex and the nap has not been concluded because of the differences in the conditions for visualization of the nap and P1 adhesin labeling by ferritin. In the present study, we used colloidal gold labeling for negative-staining EM observation. The results supported the idea of identity between the P1 adhesin and the nap structure and also indicated identity between the knoblike structures observed by ECT and the P1 adhesin complex through the observation of the class IV-22 mutant cells (Fig. 2 and 3).

The P1 adhesin complex, composed of a complex of two P1 adhesin and two P90 molecules, has been observed as a globular structure by rotary shadowing EM (21), and this complex is likely to correspond to the knoblike particles on the attachment organelle observed by ECT (Fig. 1 and 2). The knoblike particles form a relatively dense two-dimensional array, with neighboring distances ranging from 16 to 26 nm, and are present all around the tapered part of the attachment organelle, probably enabling cells to bind to host tissues in any orientation. Although we found many knoblike particles on the membrane surface of the organelle in each tomogram, we failed to obtain an averaged three-dimensional image of the knoblike particle, due to the high degree of heterogeneity in the particle images, particularly on the cytoplasmic side. This heterogeneity may suggest that the conformation changes dynamically during gliding.

### Components of the thin and thick plates.

The observations of mutant strains lacking the HMW1 (MPN447) or HMW2 (MPN310) proteins by EM (25, 31) or by fluorescence microscopy (25, 26, 32, 33) suggested that these two proteins are potentially the major components of the thin and thick plates. In the present study, we showed that these plates can change their conformation independently (Fig. 4), suggesting that the thin and thick plates are composed of different molecules, probably HMW1 and HMW2. The interdependent instability of these proteins may suggest that a structure composed of one protein supports that composed of the other (32, 34, 35).

Recently, the N and C termini of HMW2 have been shown to localize at the front and back ends of the paired plates, respectively, based on optical microscopy observation of fluorescence fusion proteins showing that the thick plate is likely composed of HMW2 (20). From the amino acid sequence, HMW2 is predicted to have 11 coiled-coil regions comprising 1,257 amino acid residues from among a total of 1,818 residues (20, 31, 36). It is possible that the striations of the thick plate could be formed by the coiled-coil regions of HMW2, because the numbers of striations ranged from 10 to 12 (Fig. 1B). However, additional experiments are needed to assign the amino acid sequence onto the thick plate. Microscopic observation of fluorescent fusion proteins also showed that HMW1 localizes at the central part of the paired plates, suggesting that the thin plate contains HMW1 (20). The amino acid sequence of HMW1, comprising 1,018 residues, can be divided into three regions (37), including an N-terminal region of 170 residues, featuring an enriched aromatic and glycine residue (EAGR) motif, a middle region of 171 to 522 residues, featuring an acidic and proline-rich (APR) region, and a C-terminal region of 523 to 1,018 residues, featuring two coiled-coil regions. The regions of the EAGR motif and coiled-coil may be involved in the lattice formation, because they are known to interact with other proteins (38).

### Possible gliding mechanism.

An inchwormlike model has been proposed for the gliding mechanism, based on the structural features revealed by ECT of the attachment organelle, in which inchwormlike conformational changes in the pair of thin and thick plates are thought to generate the propelling force (6, 16). To discuss the conformational changes involved in the gliding mechanism, detailed information on the structural periodicities of the paired plates and P1 adhesin is necessary. In the present study, we identified the probable P1 adhesin array, the periodic structures of the paired plates, and the variation in the pitch of the thick plate. This allows a more concrete mechanism for the inchwormlike model to be proposed, based on the present structural information and the data obtained by previous EM, optical microscopy, and genetic studies (Fig. 5B) (4). The knob domain of the P1 adhesin complex binds to the host cell surface through SOs (18, 21). The thick plate of the internal core is likely to extend and compress substantially by changing the periodicity of its segmented structure along the rigid thin plate. The thin plate and the proximal part of the thick plate stick together and are stably connected to the bowl structure at the back of the organelle, and the extension and compression of the distal part of the thick plate may be involved in the elongation and shortening of the entire attachment organelle. The spacing of the P1 adhesin complexes in the two-dimensional array would change accordingly, because the complexes are aligned in the limited surface space around the organelle. The putatively less-diffusive materials occupying the translucent area may be involved in coupling the movements between the core and the surface structures. By such movements, the P1 adhesin complexes repetitively attach to and detach from the surface. If the attachment and detachment of the P1 adhesin complex depends on the direction of force, with preferential detachment occurring in forward rather than backward movement, the repeated binding would result in a forward-biased displacement of the cell. The directional bias in binding and release has been suggested by the observations that mycoplasmas recognize SOs in a lock and key manner (17–19) and that the P1 adhesin complex is aligned with specific polarity on the attachment organelle. Interestingly, the P200, MPN387, and TopJ proteins, which are more related to gliding than to binding, are localized around the base of the organelle, corresponding to the bowl structure (20, 39–41). Although the direct energy source or the motor is not yet known, the force for gliding may be generated in the bowl structure and somehow conveyed to the pair of plates to cause a large conformational change of the thick plate along the stable thin plate. This mechanism is consistent with the recent observation that the lack of HMW2 decreased the gliding speed by 112-fold (42).

In the present study, we unveiled structural features that allow elucidation of the mechanism underlying the adhesion and gliding of *M. pneumoniae* and related species. However, more information is necessary to clarify this unique mechanism, including the structures, functions, roles, and behaviors of the proteins and complexes involved.

## MATERIALS AND METHODS

### Cultures.

*M. pneumoniae* M129 (ATCC 29342) and the class IV-22 mutant, which lacks P1 adhesin, P90, and P40 (29, 43), were grown in Aluotto medium at 37°C (21, 44).

### ECT data collection and tomogram reconstruction.

Quantifoil 200-mesh molybdenum grids (R 0.6/1.0; Quantifoil, Großlöbichau, Germany) were glow discharged and pretreated with a 1.5× concentrated solution of 10-nm colloidal gold particles for tomogram alignment. Three microliters of the cell suspension was applied to the grid, and the grid was blotted with filter paper and plunged into liquid ethane using a Vitrobot instrument (FEI, Eindhoven, Netherlands). Images were collected at liquid nitrogen temperature using a Titan Krios field emission gun (FEG) transmission electron microscope (FEI) operated at 300 kV on a 4-k by 4-k Ultrascan 4000 charge-coupled device (CCD) camera (Gatan, Pleasanton, CA). The pixel size on the specimen varied from 0.62 to 0.76 nm. A single-axis tilt series was collected covering an angular range from to 70° to +70° with a nonlinear Saxton tilt scheme at 8 to 10 µm under focus using Xplore3D software (FEI). A cumulative dose of 150 e^−^/Å^2^ or less was used for each tilt series. The tilt series images were generally binned using two and three-dimensional reconstructions as calculated using the IMOD software package (45). Surface-rendering images were obtained using the three-dimensional modeling software Amira 5.2.2 (Visage Imaging, San Diego, CA).

### Determination of the periodicities of the nap (P1 adhesin complex) array and the thin and thick plates.

Membrane surface areas with periodic arrays of nap structures were extracted from the tomograms. Similarly, two-dimensional sections of the thin and thick plates were extracted from tomograms where they were most visible. Extracted images of thin and thick plates were aligned and averaged using the EMAN software package (46). Then, their Fourier transforms were also calculated using ctfit from the EMAN package. The lattice parameters were determined, and the lattices were overlaid onto the images. Noise-filtered images were also computed by inverse Fourier transform.

### Immuno-EM.

Cultured cells suspended in fresh medium at 10-fold the density of the culture were placed on a carbon-coated grid and left for 10 min at room temperature. The medium was removed, and the cells on the grid were washed twice with phosphate-buffered saline (PBS; 75-mM sodium phosphate [pH 7.3], 68 mM NaCl) for 1 min each time. The cells were fixed with 3% paraformaldehyde and 0.1% glutaraldehyde in PBS for 10 min and washed twice with PBS. The cells were then treated with a primary antibody against P1 adhesin (14) at a concentration of 3 mg/ml in PBS containing 2% bovine serum albumin (BSA) (PBS-B) and then washed five times with PBS. Subsequently, the cells were treated with 10-fold-diluted gold-labeled secondary antibody (goat antibody labeled with 5-nm colloidal gold; Sigma, St. Louis, MO) in PBS-B for 30 min at room temperature, washed five times with PBS, and stained negatively with 2% (vol/vol) ammonium molybdate. The cells were then observed under an H-7000 transmission electron microscope (Hitachi, Tokyo, Japan) operating at 75 kV, and images captured by a Fast Scan-F214 CCD camera (2 k by 2 k with 14-micron pixel size; TVIPS, Gauting, Germany).

## SUPPLEMENTAL MATERIAL

Video S1 Real-time video of *Mycoplasma pneumoniae* gliding. Phase-contrast microscopy. The height of the video image is 32 µm. Download Video S1, MPG file, 14.1 MB

Video S2 Three-dimensional images of the attachment organelle, showing the nap particle array on the membrane surface and a pair of thin and thick plates forming the internal core, reconstructed from ECT. This video is related to Fig. 1. Download Video S2, MOV file, 4.5 MB

Video S3 Video of the knoblike structures forming a dense array all around the tapered protrusion of a bifurcated attachment organelle, reconstructed from ECT. This video is related to Fig. 2G. Download Video S3, MOV file, 7.8 MB

Video S4 Three-dimensional images of the internal structure of the attachment organelle, showing a pair of thin and thick plates featuring periodic patterns. This video is related to Fig. 4A and B. Download Video S4, MOV file, 8.3 MB

Video S5 The long distal part of the thick plate of the internal core going through substantial extension and compression by changing the periodicity of its segmented structure along the rigid thin plate. This video is related to Fig. 5A. Download Video S5, GIF file, 1.2 MB

Video S6 Suggested mechanism for *Mycoplasma pneumoniae* gliding. The thick plate changes its pitch and induces directed detachment of P1 adhesin from SOs, colored white, resulting in elongation of the cell protrusion, leading to cell displacement from right to left. This video is related to Fig. 5B. Download Video S6, GIF file, 4.3 MB
