# Supplementary figures and images for: Periodicity in Attachment Organelle Revealed by Electron Cryotomography Suggests Conformational Changes in Gliding Mechanism of Mycoplasma pneumoniae
Source: mBio. 2016 Apr 12;7(2):e00243-16. doi: 10.1128/mBio.00243-16 (PMC4959525; doi:10.1128/mBio.00243-16)

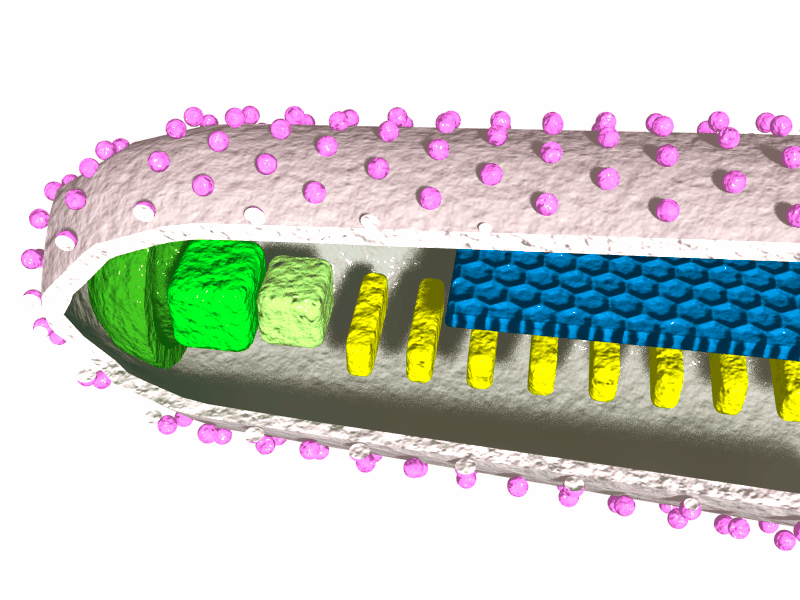

Supplement: Video S5 — The long distal part of the thick plate of the internal core going through substantial extension and compression by changing the periodicity of its segmented structure along the rigid thin plate. This video is related to Fig. 5A. Download [file mbo002162765sm5.gif]

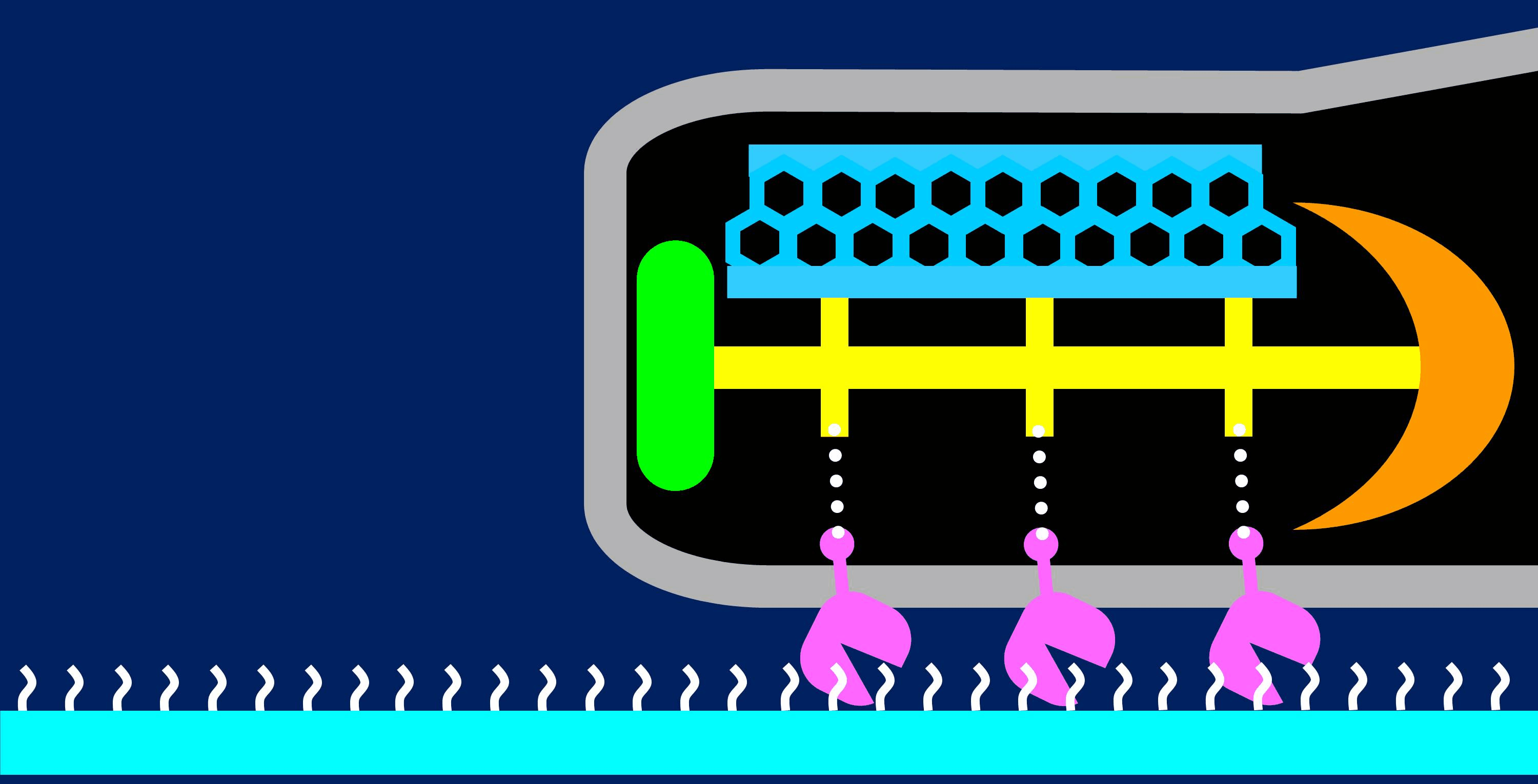

Supplement: Video S6 — Suggested mechanism for Mycoplasma pneumoniae gliding. The thick plate changes its pitch and induces directed detachment of P1 adhesin from SOs, colored white, resulting in elongation of the cell protrusion, leading to cell displacement from right to left. This video is related to Fig. 5B. Download [file mbo002162765sm6.gif]
